# Exploring the Variations in the Use of Modified Dental Anxiety Scale (MDAS) in Literature: A Survey of Studies Published from 2014 to 2023

**DOI:** 10.1002/cre2.70040

**Published:** 2024-11-13

**Authors:** Andy Wai Kan Yeung

**Affiliations:** ^1^ Oral and Maxillofacial Radiology, Applied Oral Sciences and Community Dental Care, Faculty of Dentistry University of Hong Kong Hong Kong China

**Keywords:** Bibliometric analysis, Dental anxiety, Ethics, Modified Dental Anxiety Scale, Public Health, Research, Responsible research

## Abstract

**Objectives:**

Modified Dental Anxiety Scale (MDAS) is a frequently used psychometric tool to evaluate the dental anxiety level of dental patients or the general population. However, it was largely unclear if MDAS was consistently administered in the original format in the academic literature. This work aimed to survey the literature published in the last 10 years to reveal the current usage of MDAS.

**Methods:**

Web of Science and Scopus were queried to identify papers that mentioned the use of MDAS.

**Results:**

Among a total of 260 analyzed papers, 101 papers included comprehensive information regarding both the questions posed and the response format employed. Two papers only used an explicitly renamed MDAS with modified contents. Among the 258 papers that used MDAS that were supposed to be standardized, many discrepancies from the original version were discovered. There were only 39 papers that strictly followed the recommended scoring scheme: if a participant had a score of ≥ 19, he or she might be highly dentally anxious. Notable modifications included the use of a cut‐off score different from the original recommendation, the use of multiple cut‐off scores, modifications of the response format or descriptors, and modifications to the question items especially adding extra questions.

**Conclusions:**

These modifications would create confusion when researchers and clinicians tried to compare data across studies. Researchers are recommended to administer MDAS in its original format.

## 1 Introduction

The Modified Dental Anxiety Scale (MDAS) was introduced by Humphris et al. back in 1995 (Humphris, Morrison, and Lindsay 1995). It contains five questions:

1. If you went to your dentist for treatment tomorrow, how would you feel?
2. If you were sitting in the waiting room (waiting for treatment), how would you feel?
3. If you were about to have a tooth drilled, how would you feel?
4. If you were about to have your teeth scaled and polished, how would you feel?
5. If you were about to have a local anesthetic injection in your gum, above an upper back tooth, how would you feel?

A 5‐point Likert scale is applicable to each of these questions, ranging from (1) Not anxious, (2) Slightly anxious, (3) Fairly anxious, (4) Very anxious, and (5) Extremely anxious. The total score is a sum of all five questions, ranging from 5 to 25. In other words, if a respondent answered “not anxious” to all five questions, the total score would be 5; whereas if a respondent answered “extremely anxious” to all of them, the total score would reach the maximum of 25.

In the study by Humphris et al. in 1995 (Humphris, Morrison, and Lindsay 1995), several groups of participants, namely general dental patients and university students with a major in medicine, dentistry, or psychology, had average scores in the range of 9–12. In contrast, dental phobics, who were referred to a dental fear clinic to be clinically managed by both clinical psychologists and dentists, had an average score of 21.5. Based on the sensitivity and specificity analyses, the authors determined that a cut‐off score of ≥ 19 would indicate “a strong likelihood” of dental phobia.

Later on, Humphris and his collaborators distributed MDAS to dental patients in four cities (three in Europe and one in the Middle East) using consecutive sampling (Humphris et al. 2000). Results showed that the average MDAS score for the full sample was 11.3, and 9.3% of patients scored ≥ 19. It should be noted that in this study the threshold of ≥ 19 was referred to as the cut‐off score for “high dental anxiety.” Subsequent surveys for the United Kingdom population norms conducted in 2008 and 2009 resulted in similar mean scores of 10.4 and 10.7, respectively (Humphris et al. 2013; Humphris, Dyer, and Robinson 2009). Nowadays, the MDAS is freely available in multiple languages at the website of the University of St. Andrews (University of St. Andrews 2024). In the original English version downloaded from this website, there is a line of explanation that states that a score of ≥ 19 indicates “a highly dentally anxious patient, possibly dentally phobic.”

The name MDAS itself implied that it was derived from an earlier scale called the Dental Anxiety Scale (DAS), now better known as Corah's Dental Anxiety Scale (CDAS). The latter was invented by Corah in 1969 (Corah 1969) and contained the first four questions from MDAS only. CDAS also adopts a 5‐point Likert scale for each question but uses two sets of descriptors for the responses. For Question 1, a participant needs to choose from (1) I would look forward to it as a reasonably enjoyable experience, (2) I wouldn't care one way or the other, (3) I would be a little uneasy about it, (4) I would be afraid that it would be unpleasant and painful, and (5) I would be very frightened of what the dentist might do. For Questions 2–4, the choices are (1) Relaxed, (2) A little uneasy, (3) Tense, (4) Anxious, and (5) So anxious that I sometimes break out in a sweat or almost feel physically sick. In MDAS, these descriptors were revised to the aforementioned standardized terms, considering that participants might rank “a little uneasy,” “tense,” and “anxious” in different orders (Humphris, Morrison, and Lindsay 1995). According to Humphris and co‐workers, CDAS was modified into MDAS because the public indicated that needle injection was as fearful as the drill, and original response descriptors could be confusing for some patients (Humphris, Morrison, and Lindsay 1995). Moreover, a conversion table has been published to enable the comparison between assessment scores based on CDAS and MDAS (Freeman, Clarke, and Humphris 2007). MDAS itself also has modified versions. One famous version was designed for children and called the Modified Child Dental Anxiety Scale (MCDAS) (Wong, Humphris, and Lee 1998). MCDAS contains eight questions. The first five questions were similar (but not the same) to MDAS, and the last three additional questions asked about tooth extraction, general anesthesia, and sedation.

Ideally, researchers would provide adequate descriptions of the research tools used, and cite references properly to credit the original sources of the tools and follow the original methodology. In practice, prior literature surveys have found that discrepancies indeed existed. One such example is the Mini Mental State Exam (MMSE), a 30‐point questionnaire published by Folstein et al. in 1975 (Folstein, Folstein, and McHugh 1975). Folstein et al. recommended that an MMSE score of ≤ 20 indicated that the patient was considered “likely dementia,” whereas a score of 24–30 was considered “normal” (Folstein, Folstein, and McHugh 1975). However, a study found that researchers were using at least 10 other scoring schemes for MMSE, ranging from having a different cut‐off score (still binary) to using as many as 4 cut‐off scores (patients categorized into five groups) (Monroe and Carter 2012). Another example is the Edinburgh handedness inventory (EHI), a 10‐item questionnaire published by Oldfield in 1971 (Oldfield 1971). A participant may score from −100 to +100, and Oldfield recommended a cut‐off score of 0 to dichotomize participants into left‐ and right‐handed (Oldfield 1971). However, studies found that some researchers had modified the items in the questionnaire, changed the number of questions, revised the response format, and adopted various cut‐off scores (Edlin et al. 2015; Yeung and Wong 2022). Readers should be aware that there could be many more papers using modified tools left undetected due to their lack of explicit methodological descriptions. These alterations to the original content and interpretation schemes of psychometric tools may create reproducibility/replicability issues.

It was largely unknown if MDAS was used by researchers in accordance with its original format. Hence, this work aimed to survey the literature on the use of MDAS to evaluate if papers cited the original study of Humphris, Morrison, and Lindsay (1995) (Humphris, Morrison, and Lindsay 1995) as the original source of MDAS, and if MDAS was administered in the original form without further modifications. Due to a lack of resources, literature published during the 10‐year period of 2014–2023 was surveyed.

## 2 Methods

On 1 April 2024, the following literature searches were performed:

1. Web of Science Core Collection was queried with the following search string: “modified dental anxiety scale” (Topic) OR ((MDAS OR “modified DAS*”) AND (dent* OR anxi* OR phobi*)) (Topic). Document type was limited to Article and Language was limited to English. This search yielded 227 publications.
2. Scopus was queried with the following search string: TITLE‐ABS‐KEY (“modified dental anxiety scale”) OR TITLE‐ABS‐KEY ((“MDAS” OR “modified DAS*”) AND (dent* OR anxi* OR phobi*)). Document type was limited to Article and Language was limited to English. This search yielded 257 publications.

In short, the searches looked for any mention of MDAS in the title, abstract, and keyword fields of original articles written in English. All the 484 publication records were exported into an Excel spreadsheet. After removing duplicates, 290 papers remained. Among the 290 papers, 30 of them were excluded after screening due to these reasons: irrelevance (e.g., MDAS as an abbreviation of Memorial Delirium Assessment Scale, *n* = 19), no access to the full text (*n* = 5), not original research (*n* = 3), full text not in English (*n* = 2), and no use of MDAS or its variants for adult patients (e.g. used only child version such as MCDAS, *n* = 1). Finally, 260 papers entered the analysis.

Then, the 260 papers were manually coded for the following parameters:

(1) Whether the test questions within MDAS were listed clearly.

(2) Whether the response format was described clearly.

(3) Whether the original MDAS was used.

(3a) Whether Humphris, Morrison, and Lindsay (1995) (Humphris, Morrison, and Lindsay 1995) was cited as the source of MDAS.

(3b) Number of valid cut‐off scores used and their specifics.

(3c) What references were cited for the cut‐off scores.

(3d) Any discrepancies from the original MDAS were noticed (including pure translation into other languages).

(4) Whether a modified version of MDAS was utilized with an explicitly different name.

(4a) What references were cited as the source of the modified version.

(4b) Number of valid cut‐off scores used and their specifics.

(4c) What references were cited for the cut‐off scores.

(4d) What the exact modifications were.

This work did not involve human or animal subjects, and hence ethical approval was not applicable.

## 3 Results

**Figure 1:**
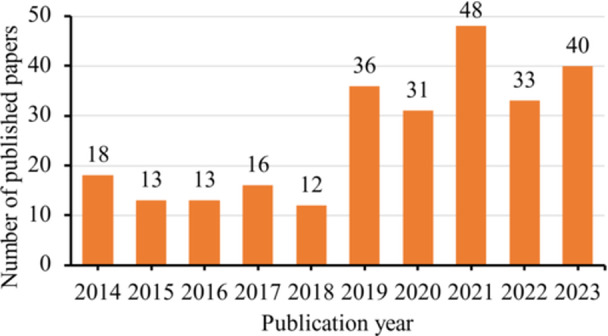
Publication year of the 260 analyzed papers.

The coded data sheet for the 260 papers was provided as Supporting File 1. There were over 30 MDAS papers published per year since 2019 (Figure 1). Overall, 258 papers reported the use of MDAS, whereas 3 papers reported the use of modified versions of MDAS with an explicitly different name. In other words, 1 paper used both MDAS and a modified MDAS. Among these 260 papers, 107 (41.2%) listed the questions clearly and 181 (69.6%) described the response format clearly. Together, 101 papers (38.8% of 260) provided details on both the questions and response format (Table 1).

**Table 1:** Breakdown of papers in terms of providing the details of the MDAS questions and response format.

		Described the response format clearly	
		Yes	No
Listed the questions clearly	Yes	101	6
	No	81	72

Next, the 258 papers that used MDAS were analyzed. Regarding giving credit to the original source of MDAS, less than half (*n* = 108, 41.9% of 258) of the papers cited Humphris, Morrison, and Lindsay (1995), with or without other references. Another 84 papers (32.6%) gave credit to other references. The remaining 66 papers (25.6%) did not cite any references as the original source of MDAS. From a different perspective where the cited references were individually considered, then other studies by Humphris et al. were also frequently cited, such as Humphris et al. (2000) (*n* = 30), Humphris, Dyer, and Robinson (2009) (*n* = 16), and Humphris et al. (2013) (*n* = 9) (Table 2). Some other commonly cited references either independently validated MDAS or translated it into other languages (Bahammam and Hassan 2014; Coolidge et al. 2008; Newton and Edwards 2005; Tunc et al. 2005) (Table 2).

**Table 2:** Commonly cited references (*n* > 5) as the source of MDAS.

Cited reference	Citation frequency (% of 258)	Comment
Humphris, Morrison, and Lindsay (1995)	108 (41.9)	Introduced MDAS
Humphris et al. (2000)	30 (11.6)	Tested MDAS in several countries, and translated it into Finnish and Arabic
Humphris, Dyer, and Robinson (2009)	16 (6.2)	Updated the MDAS data in the United Kingdom
Newton and Edwards (2005)	11 (4.3)	Tested MDAS independently
Corah (1969)	10 (3.9)	Introduced CDAS, which MDAS was based on
Humphris et al. (2013)	9 (3.5)	Updated the MDAS data in the United Kingdom
Tunc et al. (2005)	7 (2.7)	Translated MDAS into Turkish
Bahammam and Hassan (2014)	6 (2.3)	Translated MDAS into Arabic and tested in Saudi Arabia
Coolidge et al. (2008)	5 (1.9)	Translated into Spanish and tested in the United States

**Figure 2:**
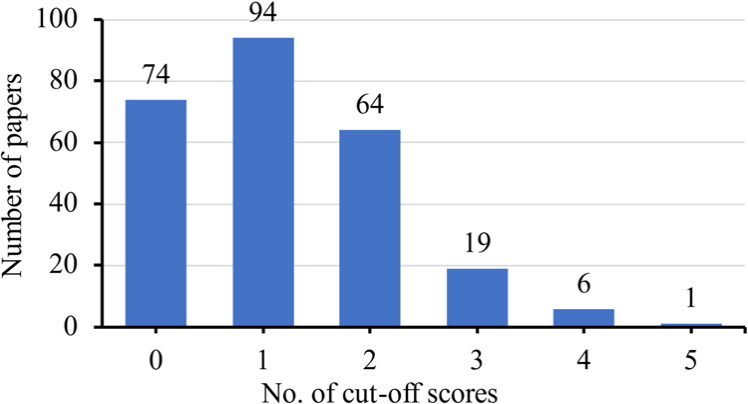
Number of cut‐off scores used by the MDAS papers.

The 258 papers most frequently used 1 cut‐off score (*n* = 94, 36.4% of 258), followed by none (*n* = 74, 28.7%) and 2 cut‐off scores (*n* = 64, 24.8%) (Figure 2). Only 31 papers (12.0% of 258) cited Humphris, Morrison, and Lindsay (1995), with or without other references, to justify their choice of the cut‐off score(s). Over half of the papers (*n* = 147, 57.0%) cited other references, whereas 80 papers (31.0%) did not cite any references for this purpose. When the cited references were individually evaluated, several papers were found to be cited frequently, including a few by Humphris (Table 3). All these references used a single cut‐off score of ≥ 19 to indicate high dental anxiety or simply dental anxiety (Humphris, Morrison, and Lindsay 1995; Humphris et al. 2000, 2013; Humphris, Dyer, and Robinson 2009; Freeman, Clarke, and Humphris 2007; İlgüy et al. 2005; King and Humphris 2010), except one that arbitrarily used 4 cut‐off scores to categorize subjects into not anxious, low, moderate, high, and extreme anxiety/phobia (Giri et al. 2017) (Table 3). It should be noted that one cited reference (Giri et al. 2017) classified subjects with a total MDAS score of 0–5 as being not anxious, which was partially impossible as the minimum total score could only be 5.

**Table 3:** Commonly cited references (*n* > 4) to justify the use of specific cut‐off score(s) for MDAS.

Cited reference	Citation frequency (% of 258)	Viewpoint
Humphris, Morrison, and Lindsay (1995)	31 (12.0)	Established that ≥ 19: High dental anxiety
Humphris, Dyer, and Robinson (2009)	13 (5.0)	Cited Humphris et al. (1995) to use ≥ 19: High dental anxiety
King and Humphris (2010)	12 (4.7)	Cited Humphris et al. (1995) and provided new data to reaffirm that ≥ 19: High dental anxiety and highly likely to have dental phobia
Humphris et al. (2013)	10 (3.9)	Cited Humphris et al. (1995) and King and Humphris (2010) to use ≥ 19: High dental anxiety
Humphris et al. (2000)	8 (3.1)	Cited Humphris et al. (1995) to use ≥ 19: High dental anxiety
Freeman, Clarke, and Humphris (2007)	5 (1.9)	Cited Humphris et al. (1995) to use ≥ 19: High dental anxiety. Provided a conversion table for researchers to convert the scores between MDAS and CDAS
Giri et al. (2017)	4 (1.6)	Cited Humphris et al. (2013) to state that ≥ 14 and ≥ 19: High dental anxiety and dental phobia, respectively. [But (3) did not mention about ≥ 14]. Translated MDAS into Nepali. Arbitrarily used 4 cut‐off scores so that 0–5: Not anxious; ≥ 6: Low; ≥ 11: Moderate; ≥ 15: High; ≥ 19: Extreme anxiety/phobia
İlgüy et al. (2005)	4 (1.6)	Cited Humphris et al. (1995) to use ≥ 19: Dental anxiety. Translated into Turkish

Among the 94 papers that used 1 cut‐off score as the original Humphris, Morrison, and Lindsay (1995) did, most of them followed the original score of ≥ 19 (*n* = 72, 76.6% of 94). Among these 72 papers, over half (*n* = 39, 54.2% of 72) defined participants who scored above this threshold as having a high level of dental anxiety. A variety of other descriptors were also used, such as having dental phobia (*n* = 10, 13.9% of 72), dental anxiety (without particular description on its level, *n* = 7, 9.7%), and extreme level of dental anxiety (*n* = 6, 8.3%) (Table 4).

**Table 4:** Distribution of the exact score and descriptors from papers (*n* = 94) that used a single cut‐off score.

Cut‐off score (in ascending order)	Number of papers	Descriptors of dental anxiety level
?	1	High
10	1	Fair
11	4	Anxiety: 2; Moderate: 2
12	1	High
13	3	High: 2; Moderate: 1
15	5	Anxiety: 4; Phobia: 1
16	3	Anxiety: 2; Phobia: 1
18	1	High/phobia
19	72	High: 39; Phobia: 10; Anxiety: 7; Extreme: 6; Very anxious: 4; Elevated risk, excessive, extreme/phobia, extremely high, high/phobia, severe/phobia: 1 each
20	2	Extreme: 1; Severe: 1
25	1	Phobia

**Figure 3:**
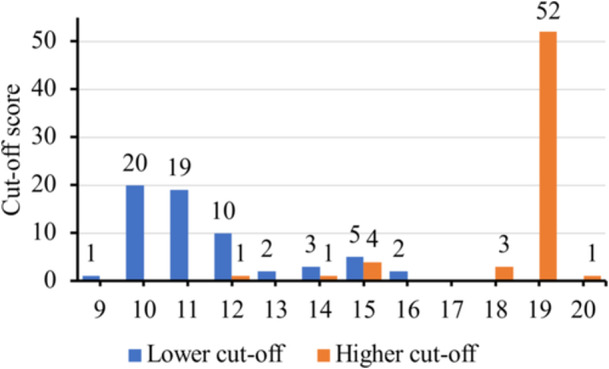
Distribution of lower and higher cut‐off scores used by MDAS papers with 2 cut‐off scores (*n* = 64). Two papers did not report the exact cut‐off scores, so the data from 62 papers were shown.

**Figure 4:**
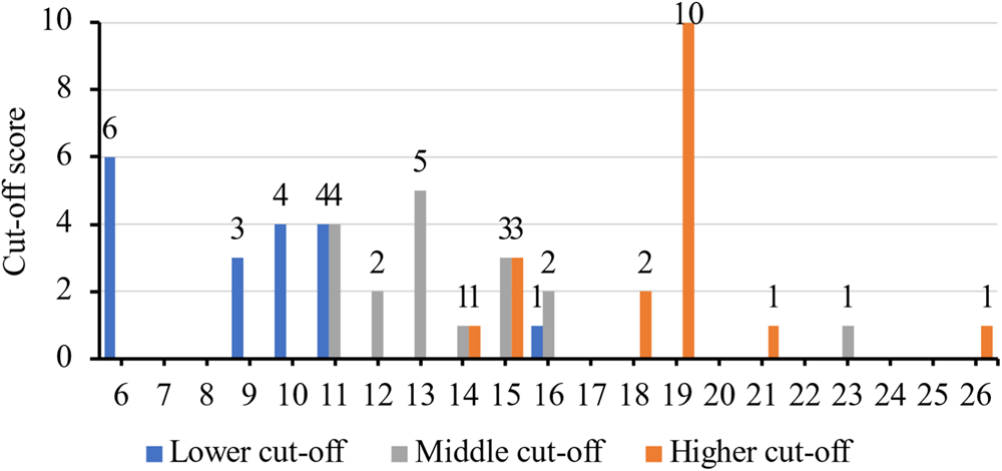
Distribution of lower, middle, and higher cut‐off scores used by MDAS papers with 3 cut‐off scores (*n* = 19). One paper did not report the exact cut‐off scores, so the data of 18 papers were shown.

For the 64 papers that used 2 cut‐off scores and 19 papers that used 3 cut‐off scores, the exact chosen scores varied hugely. For the former group of papers (n = 64), the lower cut‐off score ranged from 9 to 16 whereas the upper cut‐off score ranged from 12 to 20 (Figure 3). They usually used ≥ 10 or ≥ 11 as the lower cut‐off with the most common descriptor being a moderate level of dental anxiety. Meanwhile, the overwhelming choice for the upper cut‐off was ≥ 19 with a variety of descriptors. For the latter group of papers (*n* = 19), there was no predominant number as the lower or middle cut‐off scores, whereas ≥ 19 was again the predominant choice for the upper cut‐off score (Figure 4). It could be noticed from Figure 4 that 1 paper used ≥ 26 as the upper cut‐off score, which should be impossible given that the maximum score for MDAS is 25. Upon closer examination, that paper reported the use of MDAS that consisted of the four questions from CDAS and three additional questions on the increase in dentist fee, screening and prevention procedures, and risk of COVID‐19 transmission in dental clinics, rendering a total of 7 questions and a maximum score of 35 (Table 5).

**Table 5:** The number of papers (total n = 19) that modified the contents of MDAS without renaming it.

Number of papers	Modifications
4	Added Question 6 on getting teeth extracted
4	Response descriptors were from CDAS
2	5‐point Likert scale changed from 1–5 to 0–4
2	Asked 4 questions only (unclear which question was omitted)
1	Question 1 was omitted
1	Response descriptors were from CDAS; 5‐point Likert scale became 4‐point; Added questions on teeth being looked, filled, and extracted; Deleted Question 2
1	5‐point Likert scale changed from 1–5 to 0–4; Asked 10 questions without question details
1	Added 3 questions on getting teeth extracted, seeing instruments for extraction, and seeing blood/blood‐stained objects
1	Used the 4 questions from CDAS, and added 3 questions on the increase in dentist cost, screening and prevention procedures, and risk of COVID‐19 transmission in dental clinic
1	Question 1 changed to prepare to see a dentist at home; Question 3–5 changed from imaginary situations to recall past experience; Question 4 changed from teeth being “scaled and polished” to “treated”
1	The assessed dental situations became the first visit, in the waiting room, during dental trimming, during scaling, and during anesthesia

Abbreviation: CDAS, Corah's Dental Anxiety Scale.

In contrast to the heterogeneity of papers described above, the 6 papers that used 4 cut‐off scores were highly consistent. All of them used the following cut‐off scores: ≥ 6 (low, one paper used the descriptor “somewhat”), ≥ 11 (moderate), ≥ 15 (high), and ≥ 19 (extreme, two papers used the descriptor “extreme/phobia”). Four of the six papers cited (Giri et al. 2017) to justify the exact scoring scheme, one cited (Humphris, Dyer, and Robinson 2009) (it should be noted that (Humphris, Dyer, and Robinson 2009) only used 1 cut‐off score of ≥ 19), and one did not cite any references. Finally, 1 paper adopted 5 cut‐off scores without citing any references: ≥ 6 (extremely low), ≥ 8 (low), ≥ 11 (moderate), ≥ 16 (high), and ≥ 19 (phobia).

There were 74 papers (28.7% of 258) that translated MDAS into non‐English languages without modifying the contents. Meanwhile, 19 papers reported some modifications of the contents of MDAS but still called it MDAS. The most frequent modifications were adding an additional question on getting teeth extracted and adopting the response format from CDAS (each *n* = 4, Table 5). Readers can refer to Supporting File 1 for the details. Another three papers explicitly renamed the modified MDAS they have used, all of which could be considered novel without citing any prior studies that used the exact tools (Table 6).

**Table 6:** Modified and renamed MDAS versions used by the papers.

Name of the tool	Modifications	Used by
International MDAS	Added 5 questions: 1. Have you ever received any form of dental care? 2. Have you had any perceived difficult or adverse experiences with previous dental treatments within the country? 3. Have you ever had any perceived difficult or adverse experiences with previous dental treatments outside the country? 4. How do you feel about attending a current dental visit based on your past negative experience? 5. If you were told a story of someone else's difficult dental visit, how would you feel at a dental appointment? The single cut‐off score changed to ≥ 28 (severe)	Tajirian, Juarez, and Martinez (2023)
MDAS‐FIS	Response format becomes facial images	Fu et al. (2023)
MDAS‐DEP	Only Question 5 was kept, whereas the other 4 questions were changed to: 1. If you were told that one of your teeth had to be extracted, how would you feel? 2. If you were about to go to the dentist tomorrow to have your tooth extracted, how would you feel? 3. If you were sitting in the waiting room waiting for your dental extraction procedure, how would you feel? 4. If your third molar was about to be removed through a surgical procedure, how would you feel?	Maulina, Nadiyah Ridho, and Asnely Putri (2019)

Abbreviations: DEP, dental extraction procedure; FIS, facial image scale.

Lastly, some rare but inaccurate descriptions of MDAS were noticed in the analyzed papers (Supporting File 1). For example, three papers correctly described that the total MDAS score ranged from 5 to 25, but either mentioned that a score of 0–5 was “not anxious” or 0–10 was “good”. Another two papers correctly described that the total MDAS score ranged from 5 to 25, but then mentioned that a score of < 5 meant that the participant had no or low dental anxiety.

## 4 Discussion

This study has several major findings regarding the use of MDAS in the literature. The primary finding of this study revealed that 38.8% of the 260 analyzed papers included comprehensive information regarding both the questions posed and the response format employed. Initially, this finding may appear unimportant, given the common assumption that researchers would uniformly adopt a standardized tool when employing MDAS. However, closer examination revealed the variability of tool utilization among researchers. The most notable variations were the use of a cut‐off score different from the original recommendation, the use of more than 1 cut‐off score, modifications of the response format or descriptors, and modifications to the question items especially adding extra questions. The original scoring scheme of MDAS was straightforward: if a participant had a score of ≥ 19, he or she might be highly dentally anxious, possibly dentally phobic (University of St Andrews 2024). In this survey, only 39 papers (15% of 260) strictly followed this scoring scheme.

It is understood that different populations, for example, those in different countries or continents, may have variations in their cultural backgrounds and dental perceptions. As such, the universal application of the single cut‐off score of ≥ 19 might not fit the purposes of all studies. Hence, it was understandable that some studies established their own customized cut‐off score based on their sample. For example, Al‐Nasser et al. (2016) translated MDAS into Arabic, collected data from 64 dental or medical patients in Saudi Arabia, and compared it against their self‐reported history of missing dental appointments due to anxiety. Receiver operating characteristics (ROC) analysis showed that a cut‐off score of ≥ 13 had optimal sensitivity and specificity. Similarly, Bahammam and Hassan (2014) also translated MDAS into Arabic, collected data from 474 dental patients in Saudi Arabia, and compared against their self‐rated anxiety level in the format of a visual analog scale (VAS) taken at the waiting area before their dental appointment. Patients were classified as dental phobic if their VAS score was ≥ 70%. ROC analysis showed that a cut‐off score of ≥ 16 had the optimal sensitivity and specificity. Kassem El Hajj et al. (2021) also translated MDAS into Arabic, collected data from 451 dental patients in Lebanon, and compared it against their self‐rated anxiety levels following a similar methodology as Bahammam and Hassan (2014). (VAS score ≥ 51% was anxiety, whereas > 70% was phobia). Results found that the optimal cut‐off was ≥ 12 for dental anxiety and ≥ 14 for dental phobia. Meanwhile, Tunc et al. (2005) translated MDAS into Turkish, collected data from 115 dental patients, and compared it against their status of dental phobia. Patients were classified as dental phobic if they displayed phobic behavior and could only accept treatment under general anesthesia. Otherwise, they were classified as normal. For this Turkish sample, ROC analysis showed that a cut‐off score of ≥ 15 had optimal sensitivity and specificity.

The numerous examples discussed above clearly showed that researchers were consistently using ROC analysis to determine the optimal cut‐off score(s), but they were using very different gold standards or ground truth. It could be a self‐reported history of dental avoidance, VAS rating, or the need to undergo general anesthesia for dental treatment. Hence, the theoretical meaning and practical implications of the status of dental anxiety determined by these cut‐off scores could be largely different. In particular, it is worthwhile to have a brief discussion here on the studies that used VAS rating to validate their proposed MDAS cut‐off scores. Two studies used the VAS rating as a benchmark for validating the MDAS cut‐off scores (Bahammam and Hassan 2014; Kassem El Hajj, Fares, and Abou‐Abbas 2021). Both cited Facco et al. (2011) and stated that the latter had validated the use of VAS to assess dental anxiety, and therefore VAS could be used as a benchmark. Interestingly, Facco et al. (2011) actually validated the VAS rating by using the CDAS score as the benchmark. The original paper by Corah (1969) did not introduce any cut‐off scores for CDAS, but a subsequent study conducted by his team elaborated in the discussion section that “a score of 13 or 14 should make the dentist suspicious that he is dealing with an anxious patient. A score of 15 or more almost always indicates a highly anxious patient” (Corah, Gale, and Illig 1978). Without citing any references to justify the choice of the cut‐off scores, Facco et al. (2011) chose CDAS scores of ≥ 13 and ≥ 16 to categorize patients into having dental anxiety and dental phobia, respectively. After relating VAS and CDAS scores from 1114 dental patients by ROC analysis, they came up with the thresholds of VAS scores ≥ 51% and ≥ 70% for dental anxiety and dental phobia, respectively. Therefore, studies that used VAS scores of ≥ 51% and ≥ 70% to determine/validate customized cut‐off scores for MDAS might involve circular reasoning to a certain extent, which meant: “if A, then B” followed by “if B, then A”. It can be argued that CDAS and MDAS are not identical, nevertheless, this is a point that future studies should consider when deciding the benchmarking method.

It seems that the term “dental phobia” does not have a universally agreed definition. It could be a disproportionately intense fear that leads to avoidance of dental treatment (Freeman 1999; Lautch 1971) or as one end of a continuum of dental anxiety (Beaton, Freeman, and Humphris 2014; Wong et al. 2022). If so, it might be very difficult to obtain MDAS data from dental‐phobic patients in an ordinary clinical setting, as these patients might be too afraid to show up. While the original MDAS form states that a score of ≥ 19 indicates “a highly dentally anxious patient, possibly dentally phobic” (University of St Andrews 2024), the current survey revealed that a single cut‐off score for dental phobia could range from ≥ 15 to ≥ 25, equivalent to 60% to 100% of the maximum score (Table 4). It could be debatable whether these patients, all classified into the so‐called dental phobia group, would be homogeneous or comparable to one another. Besides, meta‐analyses would record the proportion of subjects with dental phobia reported from individual studies to compute an overall prevalence rate. Facing these variations, researchers had to set clear rules during data processing. For example, a recent meta‐analysis computed the global prevalence of dental fear, high dental fear, and severe dental fear (Silveira et al. 2021). However, they only considered 2 cut‐off scores in processing data from MDAS studies: ≥ 10 was dental fear and ≥ 19 was high dental fear. In other words, even if an original study used a cut‐off of 25 to categorize patients with dental phobia and reported a percentage, this meta‐analysis would still treat these patients as having high dental fear. Readers should also remember that many MDAS studies used multiple cut‐off scores, and there are many psychometric tools to assess dental anxiety besides MDAS. (Readers are referred to (Chi 2023; Armfield 2010) for comprehensive reviews on these psychometric tools.) Therefore, researchers and clinicians should be highly aware of the high heterogeneity between studies regarding the cut‐off scores and the descriptors of the scores regarding dental fear, anxiety, or phobia. In particular, it should be emphasized here that dental phobia can only be identified via a detailed psychiatric interview conducted by a qualified psychiatrist or psychologist. The diagnosis should be made with reference to the diagnostic criteria of phobic disorders listed in the Diagnostic and Statistical Manual of Mental Disorders, Fifth Edition (DSM‐5). Alternatively, some researchers would ask an additional set of questions to classify dental phobics instead of solely relying on the MDAS score (King and Humphris 2010).

This study has some limitations. First, only papers that utilized MDAS were evaluated, making it impossible to estimate the prevalence of MDAS usage in the literature (i.e., its literature share). Second, this study did not extract the mean overall score or the scores for individual questions from the reviewed studies. Third, this study did not differentiate how MDAS was employed in each study. For instance, MDAS might have been used to categorize subjects into different intervention groups, or simply as a criterion for including or excluding subjects during recruitment. There is a possibility that the purpose of using MDAS could influence how it was applied, which was not investigated in this work.

In conclusion, MDAS was a frequently used tool to assess the level of dental anxiety of dental patients or the general population. Among the 260 analyzed papers, 38.8% included comprehensive information regarding both the questions posed and the response format employed. Many discrepancies from the original version were discovered, with the most notable ones being the use of a cut‐off score different from the original recommendation, the use of more than 1 cut‐off score, modifications of the response format or descriptors, and modifications to the question items especially adding extra questions. All these would create confusion when researchers and clinicians tried to compare data across studies. Therefore, researchers are recommended to administer MDAS in its original format or use other tools if MDAS does not fit the purpose. Researchers should not simultaneously modify MDAS but still call it MDAS.

## Author Contributions

The sole author is responsible for all parts of the work.

## Ethics Statement

The author has nothing to report.

## Consent

The author has nothing to report.

## Conflicts of Interest

The author declares no conflicts of interest.

## Supporting information


**Caption for Supplementary File 1**: Coded data sheet for the 260 papers.

## Data Availability

Data are provided in the manuscript and Supplementary Information.
